# P01-17 Promotion of health-enhancing physical activity in Europe: a cross-sectional study among 536 sports associations

**DOI:** 10.1093/eurpub/ckac095.017

**Published:** 2022-08-29

**Authors:** Tena Matolić, Danijel Jurakić, Hrvoje Podnar, Ivan Radman, Željko Pedišić

**Affiliations:** 1 Laboratory for Physical Activity Epidemiology and Promotion, University of Zagreb, Faculty of Kinesiology, Zagreb, Croatia; 2 Department of General and Applied Kinesiology, University of Zagreb, Faculty of Kinesiology, Zagreb, Croatia; 3 Institute for Health and Sport, Victoria University, Melbourne, Australia

**Keywords:** Sports Club for Health, SCforH, sports associations, sports organisations, sport promotion

## Abstract

**Background:**

Sports associations may play an important role in the promotion of health-enhancing physical activity (HEPA) in Europe. However, no recent findings on their commitment to HEPA promotion are available. Therefore, we aimed to determine the level and correlates of the commitment of European sports associations to HEPA promotion.

**Methods:**

Representatives of 1717 sports associations from 36 countries were invited to take part in a survey conducted within the Sports Club for Health (SCforH) 2015-17 project, and 536 (31%) agreed to participate. The participants were asked about their organisation's awareness of SCforH guidelines and its commitment (0-10) to the promotion of: elite sports; health-enhancing sports; health-enhancing exercise, and other types of HEPA. An overall HEPA promotion score was calculated as the arithmetic mean of the latter three. A multiple regression analysis was conducted with the overall HEPA promotion score as the outcome variable and organisation type (“national association of a specific sport” as the reference group [ref], “European sports federation”, “national umbrella sports organisation”, “national Olympic committee”, “national sport-for-all organisation”), headquarters in an EU member state (“no” [ref], “yes”), region of Europe (“Western” [ref], “Central-Eastern”, “Northern”, “Southern”), commitment to elite sports (“low” [ref], “medium”, “high”), and awareness of SCforH guidelines (“no” [ref], “yes”) as explanatory variables.

**Results:**

The commitment to HEPA promotion was low (score: 0-3) in 32.1% (95% confidence interval [CI]: 28.1, 36.0), medium (4-6) in 39.7% (95% CI: 35.6, 43.9), and high (7-10) in 28.2% (95% CI: 24.4, 32.0) of sports associations. Being a national sport-for-all organisation (β = 1.68; 95% CI: 0.74, 2.62; *p* > 0.001), national Olympic committee (β = 1.48; 95% CI: 0.41, 2.55; *p* = 0.007), located in Central-Eastern Europe (β = 0.56; 95% CI: 0.01, 1.12; *p* = 0.047), and aware of the SCforH guidelines (β = 0.86; 95% CI: 0.35, 1.37; *p* > 0.001) was associated with greater commitment to HEPA promotion.

**Conclusion:**

Relatively low commitment of European sports associations to HEPA promotion may be increased by raising awareness of the SCforH guidelines and by considering national sport-for-all organisations, national Olympic committees, and organisations in Central-Eastern Europe as role-models in this endeavour.

